# NOD1 modulates chronic obstructive pulmonary disease progression via FOXA1/NLRP3-mediated regulation of pyroptosis

**DOI:** 10.3389/fimmu.2026.1800191

**Published:** 2026-04-17

**Authors:** Wenzhi Xie, Hui Xu, Hong-mei Shu

**Affiliations:** 1 Geriatric Department of Anqing Municipal Hospital, Anqing, Anhui, China; 2 Department of Internal Medicine (Respiratory Medicine), Wannan Medical University, Wuhu, Anhui, China; 3 Department of Pulmonary and Critical Care Medicine, Anqing Municipal Hospital, Anqing, Anhui, China; 4 Department of Pulmonary and Critical Care Medicine, Anhui Medical University Fifth Affiliated Hospital, Fuyang, Anhui, China

**Keywords:** chronic obstructive pulmonary disease (COPD), NOD1, pyroptosis, FOXA1-NLRP3 signaling axis, inflammatory cell death

## Abstract

Chronic obstructive pulmonary disease (COPD) is characterized by persistent airway inflammation and progressive airflow limitation. In this study, we established cellular models of COPD using bronchial epithelial cells and also constructed animal models to verify the protective effect of NOD1 knockout on lung tissue in COPD model mice, aiming to elucidate the molecular events linking increased NOD1 expression to inflammatory cell death. Transcriptomic and functional analyses revealed that NOD1 promotes pyroptosis in COPD via the FOXA1-NLRP3 signaling axis, with the PI3K-Akt pathway mediating these effects. Mechanistically, NOD1 suppresses FOXA1, leading to upregulation of NLRP3 and enhanced release of pro-inflammatory cytokines IL-18 and IL-1β. Knockdown of NOD1 alleviated pyroptosis and improved cell survival, effects reversed by NLRP3 overexpression. In conclusion, our findings identify the NOD1–FOXA1–NLRP3 axis as a key driver of inflammatory cell death in COPD, advancing our understanding of disease pathogenesis and highlighting potential molecular targets for therapeutic intervention.

## 1 Introduction

Chronic obstructive pulmonary disease (COPD)—a disease marked by airflow limitation and chronic respiratory symptoms—is the third most common cause of death (1) and is primarily driven by airway and/or alveolar damage due to cumulative exposure to toxic particles or gases, particularly from smoking (2, 3). COPD is a condition triggered by the interaction of environment and genetics, in which air pollution and smoking are significant risk factors (4).

The increasing prevalence of COPD highlights the urgent need for a deeper under-standing of its underlying biological mechanisms, particularly those that govern the inflammatory responses associated with the disease. Cigarette smoke (CS) continues to pose a significant environmental hazard for the development of COPD (5, 6).

In previous investigations, NOD1 has been found to be overexpressed in the pulmonary tissues of COPD patients, and NOD1 can activate the inflammatory response in lung macro-phages (7), but the specific molecular pathways regulating it in COPD are still under investigation.

Pyroptosis is initiated through signaling cascades that require functional inflammasomes; the classical pyroptosis pathway cuts gasdermin D (GSDMD) into GSDMD-N, which has an N-terminal domain, through Caspase-1, thereby increasing the membrane pore-forming ability and releasing inflammatory factors IL-18 and IL-1β into the microenvironment (8). In recent years, people have started to focus more on GSDME, which also contains an N-terminal domain in the Gasdermin family, but cutting GSDME’s N-terminal domain relies on activating Caspase-3 (9). Our previous study also showed that patients diagnosed with COPD had significantly elevated levels of NOD1 and GSDME when compared with non-COPD patients (10).

The purpose of this research is to develop COPD models using both laboratory animals and cultured cell lines and to explore whether there are corresponding molecular mechanisms mediated by NOD1 in the GSDME-related pyroptosis pathway involved in disease progression in COPD by utilizing transcriptomics and Dual luciferase. The findings may provide new insights for clinical precision medicine.

Studies to date have shown that nucleotide-binding oligomerization domain-containing protein 1 (NOD1) is markedly upregulated in COPD, indicating its possible pathogenic role through modulation of inflammatory processes (7). Despite these insights, the exact molecular regulatory pathways involving NOD1,particularly those that influence downstream mediators such as FOXA1 and NLRP3, remain poorly understood.

It has been demonstrated that NOD1 is an indispensable component of the inflammatory response, which is stimulated by diverse pathogen-associated molecular patterns (PAMPs) (11). Nevertheless, the actual interactions and regulatory mechanisms of NOD1, FOXA1 and NLRP3, especially in COPD, have not been examined in depth. A lack of detailed insight persists regarding how NOD1 affects the expressions of FOXA1 and NLRP3, and how such interactions eventually regulate cellular events, including pyroptosis, a type of programmed inflammatory cell death associated with inflammation and tissue injury in COPD (12). Addressing these gaps would help identify possible therapeutic targets to treat COPD and prevent its development.

In this study, cutting-edge approaches, such as RNA sequencing (RNA-Seq) transcriptomic analysis, gene enrichment analysis, differential gene expression analysis, and a series of mechanistic validation assays, including Dual luciferase, were used to study these molecular interactions. These methods permit high-throughput data collection and thorough examination of the expression patterns of all genes, enabling an elaborate examination of the regulatory networks of NOD1, FOXA1, and NLRP3 in COPD models. High-throughput RNA-Seq analysis helps identify new clues about the gene expression landscape that changes in COPD, and the findings will be further validated by the Dual luciferase and expression assays to further solidify the mechanistic explanations of how NOD1 controls downstream targets (13).

This investigation focuses on clarifying the involvement of NOD1 in the regulation of FOXA1 and NLRP3 expressions and understanding the downstream effects of such interactions on pyroptosis in COPD-affected cells. This study is expected to expand the existing knowledge on the molecular basis of COPD and possibly aid in the creation of specific therapeutic interventions that can regulate the inflammatory cascades underlying this condition by determining the molecular pathways connecting NOD1 with FOXA1 and NLRP3 (14). The results of this study could potentially yield essential information on how dysregulated innate immune responses perpetuate inflammation in COPD and provide evidence positioning NOD1 as a possible therapeutic option in the management of this chronic disease (15).

Although previous studies have confirmed the critical role of NOD1 in inflammatory responses and the progression of COPD, the mechanisms by which NOD1 regulates inflammation-related cell death via upstream signaling and transcription factor modulation remain poorly understood (16, 17). Notably, FOXA1, a key transcription factor involved in epithelial differentiation and inflammatory gene regulation, may serve as a pivotal mediator linking NOD1 signaling to NLRP3 inflammasome activation (18). Based on these findings, this study systematically explored the potential regulatory relationship between NOD1 and FOXA1, and analyzed their effects on NLRP3-mediated pyroptosis and COPD inflammatory progression. This research provides a new theoretical framework for understanding the molecular mechanisms underlying COPD pathogenesis. Importantly, our results suggest that targeted modulation of the NOD1–FOXA1–NLRP3 pathway may inhibit inflammatory responses and pyroptosis, thus offering theoretical support and novel insights for the development of precise therapeutic strategies to alleviate COPD progression.

Based on previous findings demonstrating a significant upregulation of NOD1 in COPD tissues (19), this study aims to clearly propose and validate the scientific hypothesis that “NOD1 regulates COPD progression via FOXA1/NLRP3-mediated pyroptosis.” By integrating transcriptomic analysis with molecular mechanism experiments, we systematically elucidated the role of the NOD1–FOXA1–NLRP3 signaling axis in COPD, highlighting its innovative potential as a therapeutic target. These findings provide a theoretical foundation for advancing our understanding of COPD pathogenesis and developing targeted intervention strategies.

## 2 Materials and methods

### 2.1 Animal experiments

#### 2.1.1 Ethics statement

All procedures involving animals were performed in accordance with protocols approved by the Animal Ethics Committee of Obio Metabiotechnology Co., Ltd. (IACUC 127).

#### 2.1.2 The mouse model of COPD

The chronic obstructive pulmonary disease (COPD) mouse model was established using a combination of cigarette smoke exposure and intratracheal lipopolysaccharide (LPS) instillation. Specifically, male C57BL/6 mice (aged 6 weeks, Beijing Speyford Biotechnology Co., Ltd., License No.: SCXK(Beijing) 2024-0001)) were acclimated for 7 days. On days 0 and 15, mice received intratracheal instillation of 50 μL of 1 mg/mL LPS solution. From days 1–14 and 16–29, mice were exposed daily to cigarette smoke for 60 minutes, generated by the burning of approximately three cigarettes per session within a sealed chamber. This protocol has been validated to reliably induce COPD-like pathological changes in mice (20).While control mice were conventionally housed. Prior to model induction, treatment groups received intratracheal administration of AAV-LungM3 (21) (**Supplementary Table 1**) (1 × 10^11 viral genomes/mouse in 50 μL volume) carrying either shNOD1 or an empty vector (**Supplementary Table 2**). Four weeks later, tissue samples were collected following euthanasia of the animals in a carbon dioxide chamber using a flow rate equivalent to 40% of the chamber volume per minute. Subsequently, pulmonary function in all groups was assessed using a Bellerophon RES3050 lung function test system.

#### 2.1.3 Hematoxylin and eosin staining

For staining procedures, paraffinized lung tissue slices were baked, deparaffinized, and rehydrated prior to histological staining. Following staining with hematoxylin (ZLI-9610, Zhongshan Goldenbridge) for 3–5 minutes, the sections underwent thorough rinsing using flowing tap water, differentiation using 1% hydrochloric acid in ethanol, and bluing with G1040 solution (G1040, Servicebio). Subsequently, sections were counterstained with eosin staining solution (G1100, Solarbio) for 3–5 minutes, dehydrated, cleared, and mounted. Observation and image acquisition of the stained sections were conducted with a light microscope (BX43, Olympus).

### 2.2 Cell culture and cell model construction

#### 2.2.1 Construction of NOD1 interference vector

Based on the human NOD1 transcript, shRNA targeting sequences are designed. The information regarding shRNAs is shown in **Supplementary Table 3**. The vector map of the lentivirus vector pCLenti-U6-shRNA-CMV-Puro-WPRE is shown in **Supplementary Table 4**, where the sequence for shRNA to be inserted is indicated. Lentivirus-based non-targeting shRNA and specific shRNAs for NOD1 were obtained from Obio (Shanghai corp. Ltd. China).

#### 2.2.2 Lentivirus titers

Titers (TU/mL) were calculated using the formula:(**Supplementary Table 5**).

TU/mL = (C × N × D × 1000)/V.

where C = copy number (assumed 1), N = number of cells, D = dilution factor, V = virus volume (μL).

#### 2.2.3 Cell culture

Cellverse-sourced BEAS-2B cells, a normal human bronchial epithelial cell line, were grown in DMEM (Gibco, C11995500BT) with 10% fetal bovine serum (FBS) and 1% penicillin–streptomycin (both from Gibco) at 37°C with 5% CO_2._

#### 2.2.4 Generation of stable cell lines

Lentiviral particles were prepared by transfecting 293T cells with lentiviral packaging components. The virus was collected and used to infect BEAS-2B cells with either GL401 NC3 (control vector) or Y33634 (shRNA vector). After 72 hours, infected cells were selected in puromycin-containing medium for two weeks.

#### 2.2.5 CSE model

To prepare cigarette smoke extract (CSE), the smoke from a single Marlboro Red Box cigarette (China) was bubbled into 10 mL of fresh culture medium. After a 5-minute exposure, 200μL of the solution was used to measure absorbance at 320 nm. The absorbance value of 1.0 at 320 nm corresponds to 100% CSE. After filtration with a 0.22 μm membrane, the solution was used in subsequent experiments. For example, a 4% CSE solution was prepared by diluting the stock solution (prepared as described above) with DMEM supplemented with 10% FBS and 1% PS glucose to a final volume of 1 mL (e.g., 960 μL DMEM + 40 μL fresh medium).

#### 2.2.6 Cell model construction

A total of 8,000 BEAS-2B cells were seeded into each well of a 96-well plate and incubated for 24 hours. When confluency reached approximately 70% to 80%, cells were subjected to treatment with 4% CSE and 1 μg/mL lipopolysaccharide (LPS; Sigma-Aldrich) to induce inflammatory injury. When BEAS-2B cell confluence reached 70%–80%, the cells were stimulated with 4% CSE and 1 μg/mL lipopolysaccharide (LPS; Sigma-Aldrich) for 24 hours to induce an inflammatory injury model. All experiments were performed in triplicate to ensure reliability of the results. In our cellular model, combined stimulation with CSE and LPS induces oxidative stress and inflammatory signaling, thereby promoting endogenous activation of the NOD1 signaling. This approach is intended to mimic the inflammatory microenvironment of airway epithelium in COPD patients exposed chronically to cigarette smoke and bacterial components. Experimental groups included:

1. Untreated control,
2. Model (CSE+LPS),
3. shNOD1-transduced model cells, and
4. shNC-transduced model cells.

Triplicate technical repeats and a minimum of three independent biological replicates were performed for each experiment. Phenotypic evaluation was carried out by qRT-PCR, Western blot, CCK-8 assay, and immunofluorescence as described below.

### 2.3 RNA sequencing

Total RNA from BEAS-2B cells in each experimental group was extracted using TRIzol reagent (Invitrogen, CA, USA). The NanoDrop 2000 spectrophotometer (Thermo Scientific, MA, USA) was employed to measure RNA concentration and purity. High-throughput sequencing was carried out using the Illumina NovaSeq 6000 platform under PE150 mode.

### 2.4 Gene and protein expression validation

#### 2.4.1 Quantitative RT-PCR

Total RNA was extracted from cells exposed to four varying experimental conditions (Control, CSE + LPS model, shNOD1, and shNC; n = 3 per group) using TRIzol reagent (Invitrogen,15596018) and purified with a silica column kit. RNA samples were analyzed for integrity by agarose gel electrophoresis and quantified with a NanoDrop spectrophotometer. M-MuLV reverse transcriptase with oligo (dT)_18_ primers were used to generate cDNA from total RNA.

qRT-PCR was implemented by use of SYBR Green reagents (Aidlab Biotechnologies Co., Ltd) on an ABI 7500 system. After normalization to GAPDH, relative expression levels of NOD1, FOXA1, and NLRP3 were determined by use of the 2^–ΔΔCt^ method. Primer sequence information is available in **Supplementary Table 6**.

#### 2.4.2 Western blot

RIPA buffer (Beyotime, P0013B) was employed to extract total protein, while nuclear extracts were prepared separately. Protein concentrations were determined by use of the BCA Protein Quantitation Kit (Biosharp, China). Total protein (30 µg) obtained from BEAS-2B cells was electrophoresed and transferred onto membranes. Blocking was performed with 5% BSA (Biosharp, China), followed by incubation with primary antibodies: NOD1 (Santa Cruz, sc-398398; 1:1000), FOXA1 (CST, 53528S; 1:1000), NLRP3 (Proteintech, 68102-1-Ig; 1:2000), and GAPDH (Proteintech, 60004-1-Ig; 1:50000). Finally, the proteins were visualized using ECL scanner (ChemiScope 5300 Pro).

### 2.5 Pyroptosis-related cell phenotype assay

#### 2.5.1 Experimental design

To explore the role of NOD1 in pyroptosis and its interaction with NLRP3 activation, BEAS-2B cells were distributed into the following treatment conditions:

1. Control (untreated).
2. Model (4% CSE + 1 μg/mL lipopolysaccharide [LPS]).
3. shNOD1 (Model + NOD1-targeting shRNA).
4. shNC (Model + non-targeting shRNA).
5. CY-09 (Model + 10 μM NLRP3 inhibitor).
6. shNOD1+Nigericin (Model + shNOD1 + 5 μM NLRP3 activator).

#### 2.5.2 Cell viability assays

Exponentially growing cells were seeded at a concentration of 8,000 cells per well in 96-well plates and incubated for 24 h. CSE/LPS was applied to induce injury in groups 2–6. CY-09 (10 μM) or Nigericin (5μM) was administered post-modeling as per group assignment. Each well received 10 μL of CCK-8 solution following treatment, and plates were incubated for an additional hour at 37 °C. The absorbance at 450 nm was captured using a Thermo MK3 reader, and cell viability was analyzed in reference to the control group.

#### 2.5.3 Pyroptosis-related gene expression by qRT-PCR

Following RNA extraction with TRIzol reagent, cDNA was synthesized with the TRUEscript M-MuLV Reverse Transcriptase Kit (RN3501, Aidlab Biotechnologies Co., Ltd). SYBR Green-based real-time PCR was implemented on the ABI 7500 system to evaluate gene expression. GAPDH was used as a normalization control, and relative expression levels were determined using the2^–ΔΔCt^ method. Target genes included NOD1, FOXA1, NLRP3, Caspase-1, Caspase-3, and GSDME. Primer sequences are listed in **Supplementary Table 6**.

#### 2.5.4 Western blot analysis

The study design included six groups of BEAS-2B cells exposed to different treatments: control, CSE+LPS, shNOD1, shNC, CY-09 (NLRP3 inhibitor), and shNOD1+Nigericin (NLRP3 activator). Following a 24-hour co-stimulation with 4% CSE and 1 μg/mL LPS, intervention groups received CY-09 (10 μM, 30 min) or Nigericin (5 μM, 1.5 h) post-stimulation.

Following lysis in RIPA buffer, total protein levels were measured by BCA assay. Samples containing 30 μg of protein were electrophoresed, blotted onto PVDF membranes (Millipore, HATF00010), and incubated with primary antibodies overnight at4 °C, followed by HRP-conjugated secondary antibodies (ZSGB-Bio, ZB2301,2305, 1:5000) (37 °C, 1h). Signals were visualized using ECL reagents (Dingguo, Beijing) and captured on the ChemiScope 5300 Pro imaging system.

#### 2.5.5 Enzyme-linked immunosorbent assay

Quantification of IL-1β and IL-18 in collected culture supernatants was carried out using ELISA kits (EHC002b and EHC127; Sinobest Biotech, China) in strict accordance with the manufacturer’s guidelines. A standard curve was constructed in parallel with sample testing. Biotin-labeled antibodies and HRP-conjugated detection components were applied in sequence, followed by TMB-mediated color development and acid-based termination of the reaction. Absorbance readings at 450 nm were acquired by use of a microplate reader (Multiskan™ FC, Thermo Fisher). Cytokine concentrations were calculated using a four-parameter logistic (4PL) curve-fitting model. All assays were performed in triplicate.

#### 2.5.6 Immunofluorescence staining

BEAS-2B, BEAS-2B+shNOD1, and BEAS-2B+shNC cells were seeded onto sterile glass coverslips (2 × 10^5^ cells/well) in 12-well plates and treated as described previously. Post-stimulation with CSE+LPS, CY-09 or Nigericin was applied according to the grouping in the ELISA experiment. After fixation with 4% paraformaldehyde, cells were blocked using a 5% FBS solution in PBS and incubated with anti-NOD1, anti-FOXA1, or anti-NLRP3 antibodies at 4 °C overnight. The antibodies were obtained from Santa Cruz (sc-398398), CST (53528S), and Proteintech (68102-1-Ig), respectively. PBS-washed cells were exposed to Cy3-conjugated secondary antibodies (Beyotime) and incubated at 37 °C for 1 hour, protected from ambient light to preserve fluorescence. Nuclear staining was performed using Hoechst 33342. Coverslips were mounted and imaged using an Olympus IX73 inverted fluorescence microscope under identical exposure settings across all groups.

### 2.6 Statistical analysis

All statistical analyses were performed according to standardized procedures to ensure reliability and reproducibility of data processing. RNA-Seq data underwent rigorous quality control followed by normalization, and gene expression changes were assessed using established differential expression algorithms. Multiple comparisons were corrected using the false discovery rate (FDR) method to control for false positives, and selection criteria for differentially expressed genes were based on both statistical significance and fold-change thresholds. Enrichment analyses, including GO and KEGG, were considered significant at FDR < 0.05.

Experimental data are presented as mean ± standard deviation (SD). Statistical analyses were conducted using GraphPad Prism software. Differences between two groups were assessed by independent samples t-test, while comparisons among multiple groups utilized one-way analysis of variance (ANOVA) with appropriate *post hoc* tests determined by data distribution. Statistical significance was defined as p < 0.05.

## 3 Result

### 3.1 Pulmonary function tests

**Figure 1:**
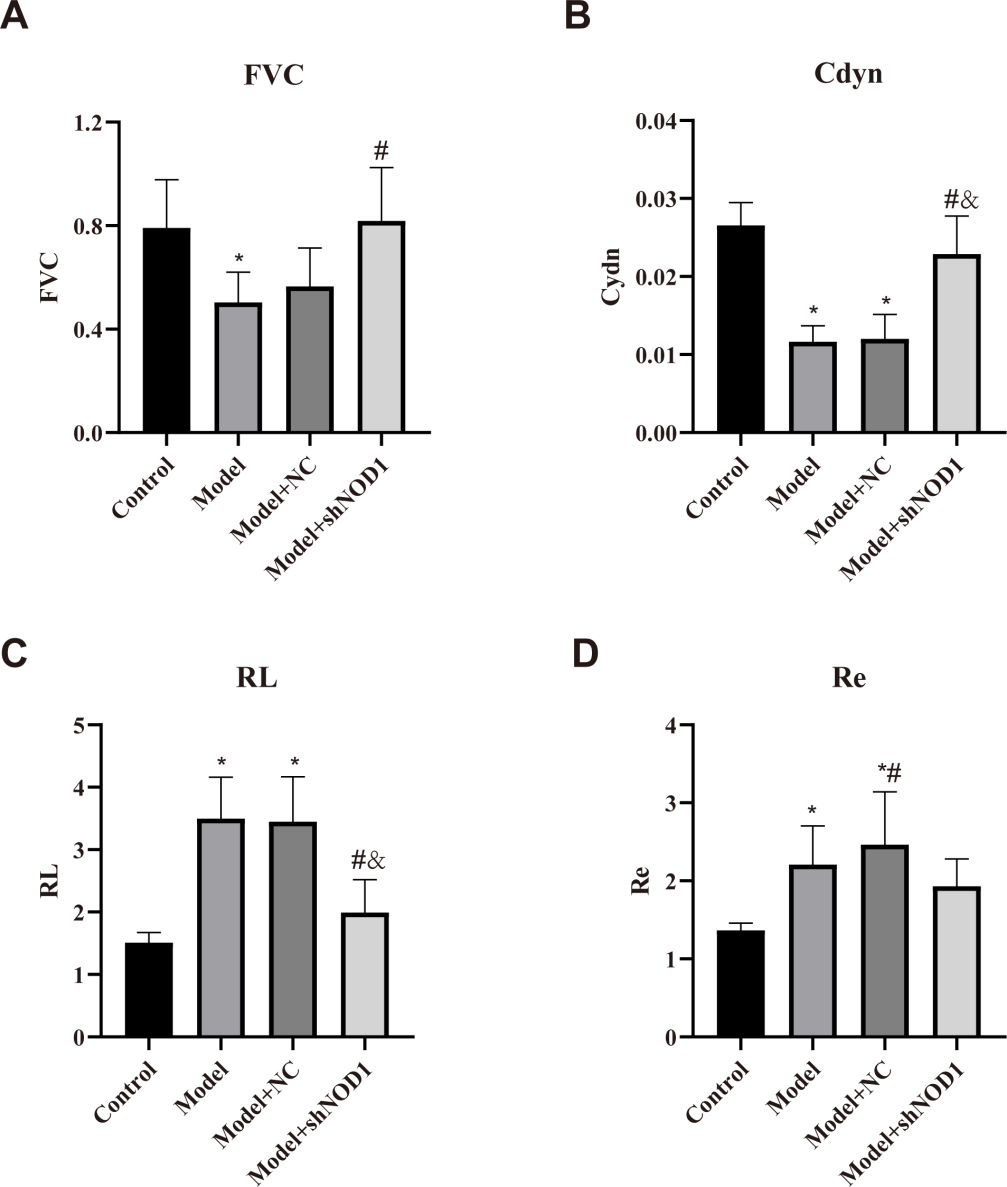
Pulmonary function tests. Compared to controls, the Model group showed reduced FVC and Cdyn, and increased Ri and Re (p < 0.05). The Model+shNOD1 group exhibited significantly higher Cdyn and lower Ri versus Model+NC (p < 0.05). (*p < 0.05 vs. Control group; #p < 0.05 vs. model group; &p < 0.05 vs. model+sh-NC group.).

Each group of mice was evaluated in terms of pulmonary function. Significant reductions in forced vital capacity (FVC) and dynamic lung compliance (Cdyn), along with increases in inspiratory airway resistance (Ri) and expiratory airway resistance (Re) were significantly increased in the Model group when compared with the Control group (*p* < 0.05) (**Figure 1**), which indicates that the COPD model mice had impaired lung function in the COPD model mice. Moreover, the Model+shNOD1 group had a significant increase of Cdyn and significant decrease of Ri versus the Model+NC group (*p* < 0.05). The data support that NOD1 interference has the capacity to enhance lung performance and reduce the pulmonary dysfunction in the COPD mouse model.

### 3.2 Hematoxylin and eosin staining

**Figure 2:**
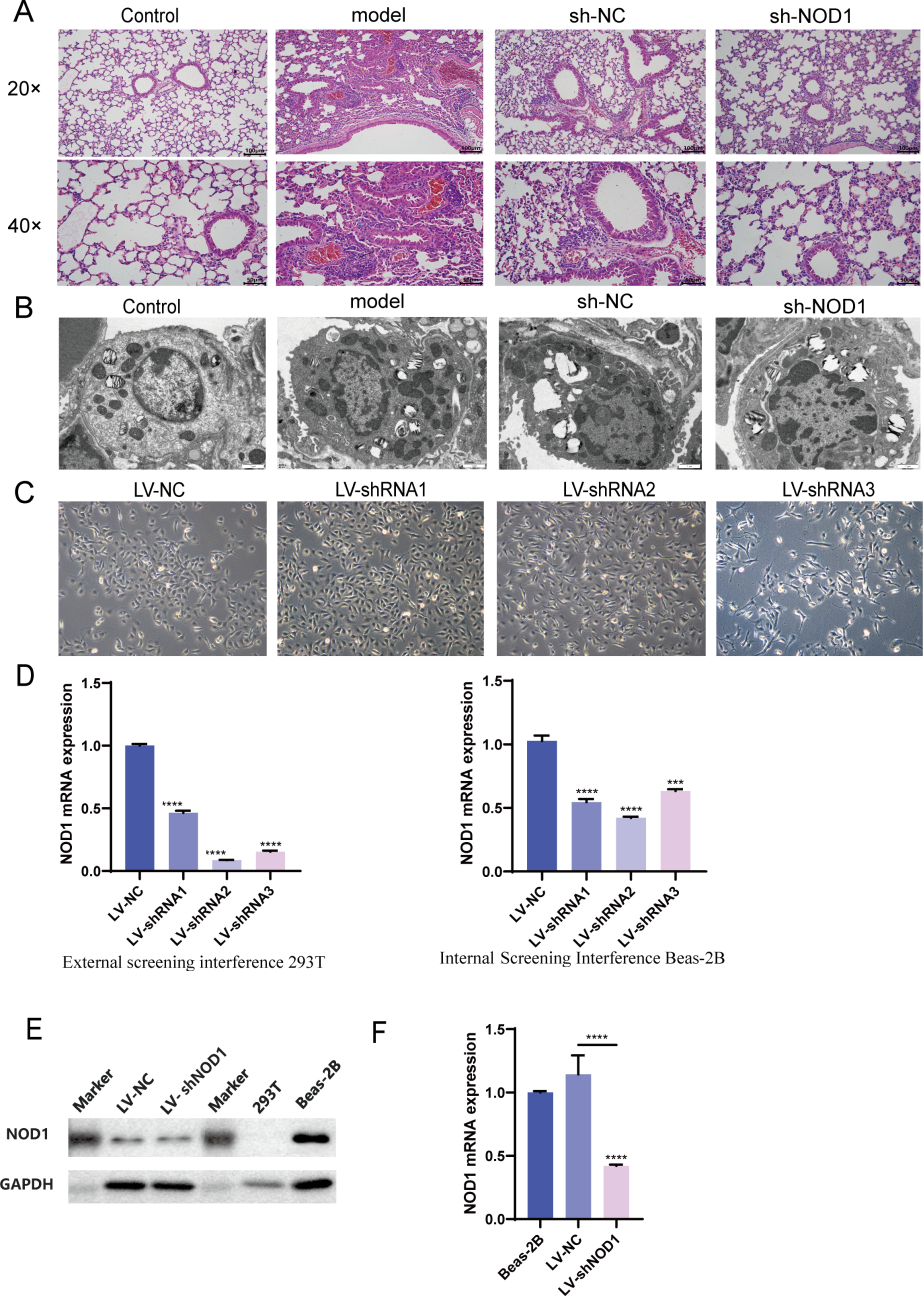
NOD1-mediated pyroptosis in bronchial epithelial cells. **(A)** Hematoxylin and Eosin (HE) Staining;Light microscopy revealed no significant histopatho- logical changes in the lung tissue of the Control group. The Model group showed marked alveolar wall thickening (blue arrows), thickened vascular and bronchial walls (yellow arrows), and dense inflammatory cell infiltration in the interstitium (black arrows). In the sh-NC group, abundant interstitial inflammatory cell infiltration (black arrows) and bronchial epithelial shedding (red arrows) were observed. In contrast, the shNOD1 group exhibited reduced inflammatory infiltration, with no obvious thickening of the alveolar or bronchial walls. **(B)** Transmission electron microscopy (TEM), Comparative images of the ultrastructure of animal tissue cells ; Control group: Cells displayed intact structure with normal nuclei and organelles, without pathological changes. model group: Notable ultrastructural damage was evident, including swollen and disorganized mitochondria and disrupted cellular architecture. shNOD1 group: Cellular morphology was substantially improved compared to the Model group, showing reduced pathological changes after NOD1 silencing. shNC group: Ultrastructural damage was similar to the Model group, indicating specificity of the protective effect to NOD1 silencing. Lentivirus-mediated NOD1 stable clone selection and validation: **(C)** Post-infection:Lentivirus-mediated interference of BEAS-2B cells revealed that shRNA3 slightly affected cell proliferation, while other targets showed no significant impact on cell growth; **(D)** External and internal screening interference 293T:Lentivirus-mediated effective target cell selection for validated targets **** p<0.001 LV-shRNA 1-3 vs LV-NC; **(E)** Westernblot: Validation of Interference in stable clones; **(F)** qPCR validation of interference in stable clones **** p<0.001 LV-shNOD1 vs LV-NC.

As shown in **Figure 2A**, light microscopic examination revealed that the Control group maintained normal pulmonary structure, with no visible pathological disruptions. Prominent alveolar wall thickening (blue arrows) was observed in the Model group, together with structural thickening of bronchial and vascular walls (yellow arrows) and dense infiltration of inflammatory cells within the pulmonary interstitium (black arrows). Similar to the Model group, the sh-NC group exhibited heavy infiltration of inflammatory cells across the pulmonary interstitium (black arrows), along with evidence of bronchial epithelial cell shedding (red arrows). Histological analysis of the shNOD1 group revealed decreased inflammation and no visible hypertrophy of the alveolar or bronchial walls, reflecting a potential therapeutic benefit.

### 3.3 Transmission electron microscopy

As shown in **Figure 2B**, transmission electron microscopy (TEM) images show comparative images of the ultrastructure of animal tissue cells. Control group: The cellular structure was intact, with regular nuclear morphology and normal organelle appearance such as mitochondria. No obvious pathological features were observed, representing the ultrastructure of tissue cells under physiological conditions.

Model group: Numerous abnormal intracellular structures were observed, including swollen and disorganized mitochondria, along with compromised integrity of the overall cellular architecture, indicating significant pathological damage.

shNOD1 group (NOD1 gene silencing): The pathological damage in cells was markedly alleviated compared to the Model group, with cellular nuclei and organelles exhibiting morphology closer to the normal state. This suggests that silencing of the NOD1 gene may have an ameliorative effect on cellular pathological changes.

shNC group (negative control): The degree of cellular structural damage was similar to that of the Model group, indicating that the effect of shNOD1 was specifically attributable to NOD1 gene silencing rather than off-target effects of the interference sequence itself.

### 3.4 Lentivirus-mediated NOD1 stable clone selection and validation

To test the hypothesis that decreased NOD1 expression is implicated in COPD pathophysiology, a Lentiviral construct (pCLenti-U6-shRNA3CMV-Puro-WPRE) encoding shRNA3 that most effectively silences NOD1(**Figure 2C**). Lentivirus-mediated infection of BEAS-2B and 293T cells revealed, via qPCR, that shRNA2 exhibited high interference efficiency (**Figure 2D**). Subsequent selection of shRNA2 was performed for further study. The NOD1 knockout was confirmed using Western blot and qPCR, which suggest NOD1 stable clone is successfully established (**Figures 2E, F**).

### 3.5 Transcriptome data

Research has demonstrated that NOD1 expression is significantly elevated in COPD patients (7). However, the precise molecular regulatory mechanisms by which NOD1 contributes to COPD remain incompletely elucidated. To investigate these mechanisms, we utilized CSE to treat BEAS-2B cells, establishing an *in vitro* model of COPD. We then employed a lentiviral vector (LV-shNOD1) to constitutively overexpress NOD1 in COPD-derived cells. The samples were grouped into the following experimental conditions: control (Ctrl), model (Model), shNC (negative control for shRNA), and shNOD1 (NOD1 knockdown). In total, 12 biological samples—three from each group—were subjected to RNA sequencing analysis.

Data preprocessing consisted of the elimination of low-quality reads, artifacts, and reads having disproportionate composition of unknown nucleotides. Further analysis showed that in all sample datasets, the Q20 values were above 97% and the Q30 values were above 92% (**Supplementary Table 7**), which showed that the constructed cDNA libraries had high sequencing quality, enabling downstream bioinformatics analysis.

**Figure 3:**
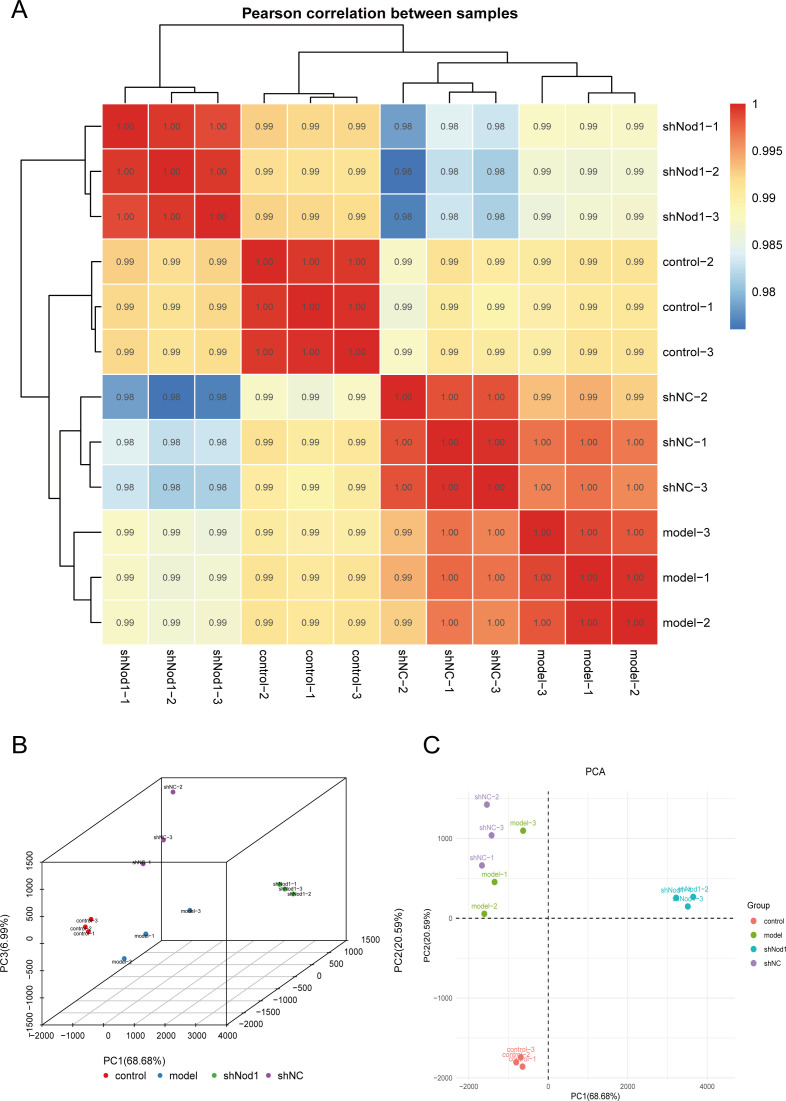
RNA-seq analysis **(A)** inter-sample correlation heatmap: The x-axis and y-axis represent samples. Numbers within the cells indicate the correlation coefficients between the corresponding samples, with darker cell colors signifying a higher correlation. **(B, C)** 2D and 3D PCA Plots:2D/3D Principal Component Analysis (PCA) Plots (In the plot, points of the same color represent individual biological replicates within each group, and the distance between points reflects the overall expression differences among samples. PC1, PC2, etc., denote different principal components, with numbers in parentheses indicating the percentage of explained variance for each component.

In order to compare patterns of gene expression between replicates, we calculated Pearson correlation coefficients. Strong correlation among all samples was observed, with coefficients greater than 0.8, supporting high experimental consistency (**Figure 3A**). Both two-dimensional and three-dimensional principal component analysis (PCA) further supported the strong clustering of RNA expression patterns in each group (**Figures 3B, C**), which is indicative of excellent replicative consistency.

### 3.6 Differential gene analysis

**Figure 4:**
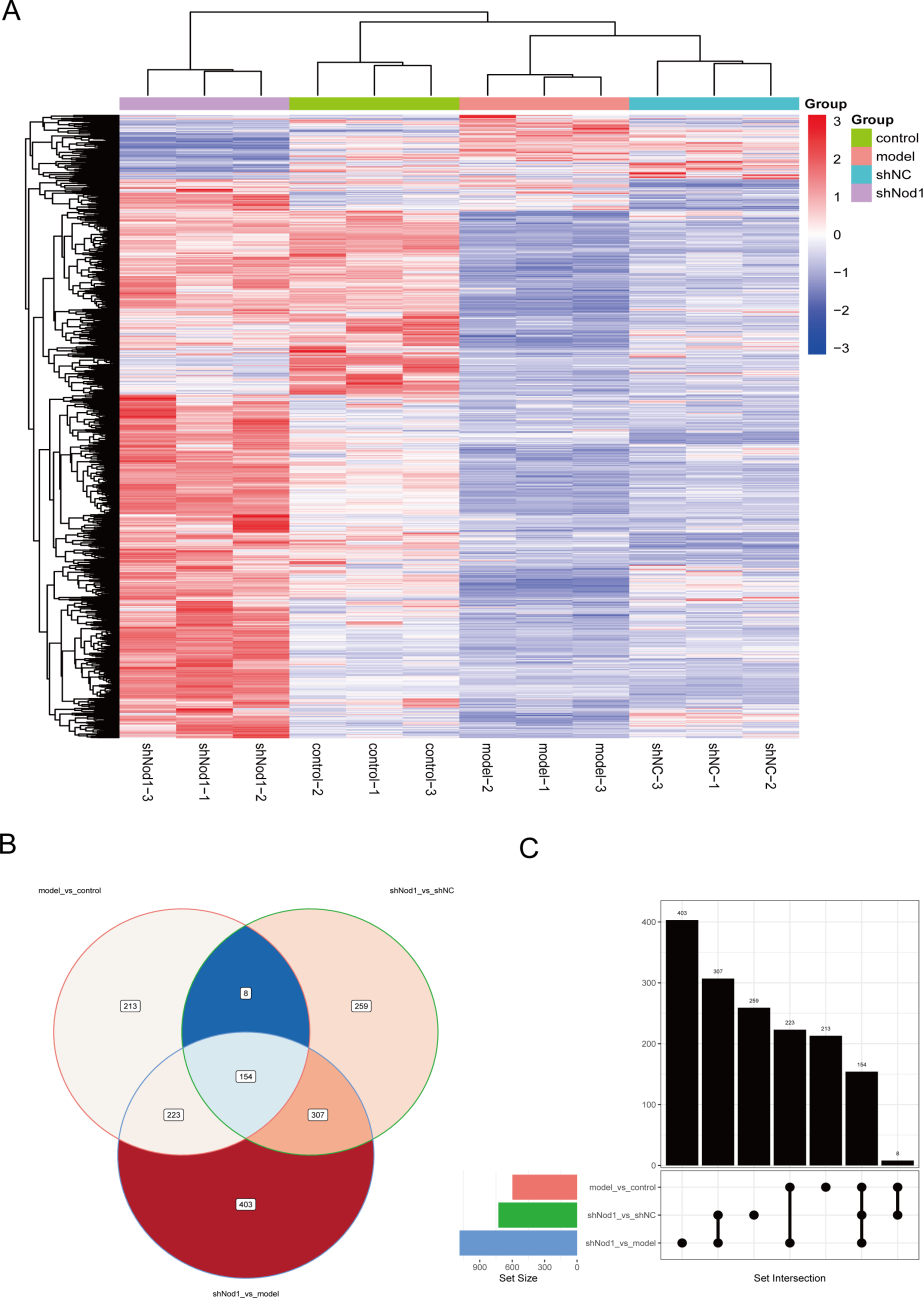
Differential gene analysis **(A)** Heatmap of all groups: Each column represents a sample, and each row represents a gene. Red indicates relatively high gene expression, while blue indicates relatively low expression. The dendrogram at the top illustrates sample clustering; closer branches between two samples indicatemore similar expression patterns of the genes displayed. Likewise, the dendrogram on the left illustrates gene clustering; closer branches between two genes signify more similar expression levels.Differential genes expressions of shNOD1 group versus that of other group ((pvalue < 0.05 and FoldChange (FC) ≥ 2); **(B)** Venn Diagram of Differentially Expressed Genes from Different; **(C)** UpSet Diagram of Differentially Expressed Genes from Different: **(B, C)** Venn diagram and UpSet plots showing that 154 genes were differentially expressed in all three comparisons.

The model group and the control group had 598 differential expressed genes (DEGs), of which 25 and 573 were upregulated and downregulated, respectively. Between the model group and shNod1 group, 1087 DEGs were detected, comprising 983 with higher expression and 104 with lower expression in the shNod1 group. Additionally, 728 DEGs were found between the shNC group and shNod1 group, with 661 DEGs upregulated and 67 DEGs downregulated in the shNod1 group (**Figure 4A**, A for heatmaps of all groups). Venn diagrams and UpSet plots revealed overlapping DEGs across the three comparison groups, showing that 154 genes were differentially expressed in all three comparisons (**Figures 4B, C**, Venn diagram and UpSet of the three comparisons).

### 3.7 Differential gene enrichment analysis

**Figure 5:**
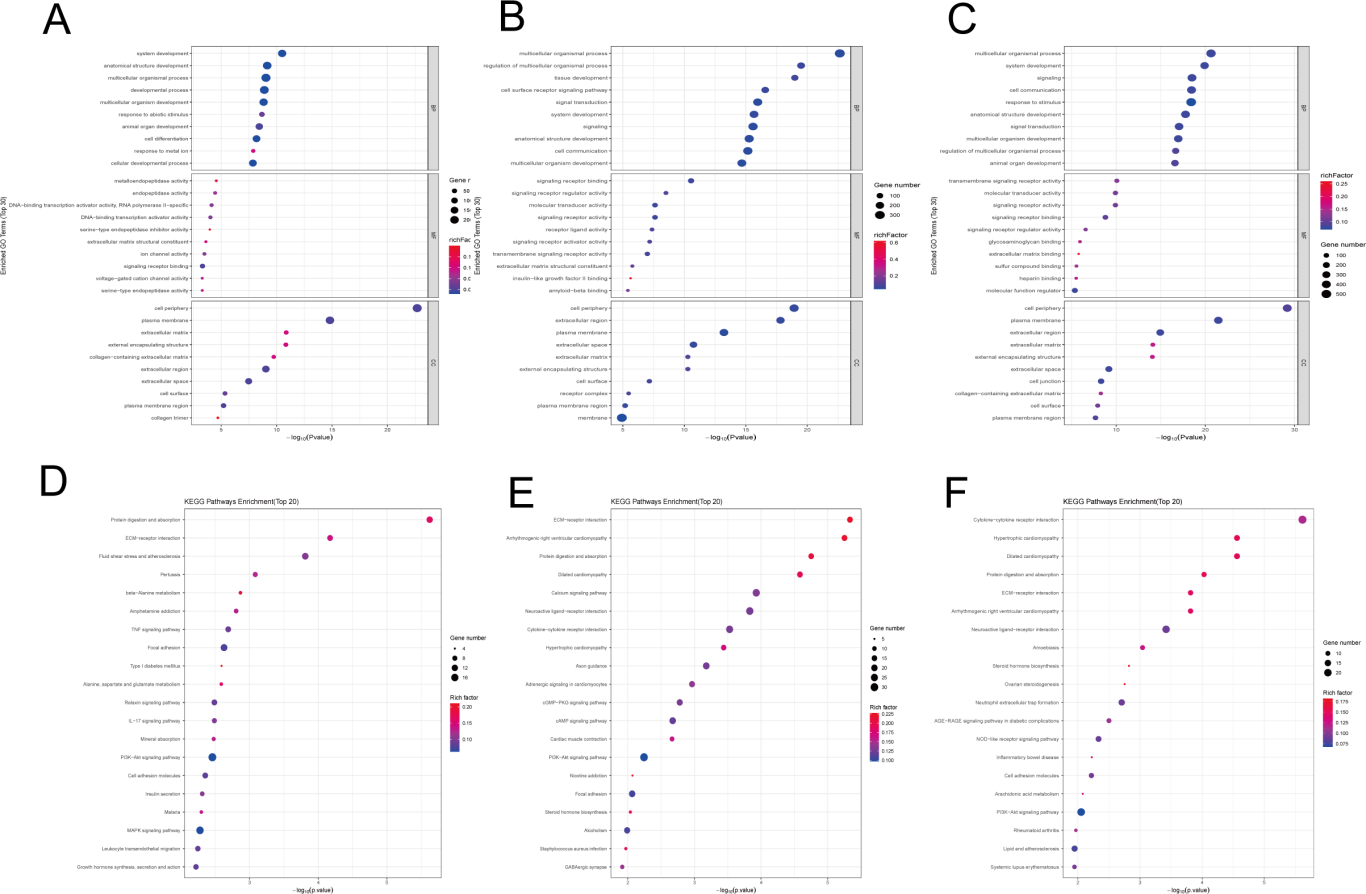
Differential gene enrichment analysis **(A–C)** GO Enrichment Bubble Plot of Differentially Expressed Genes (The x-axis represents the -log10-transformed P-value, with entries sorted in ascending order by P-value. The y-axis displays the descriptive names of the specific functional terms, categorized from top to bottom into Biological Process (BP), Molecular Function (MF), and Cellular Component (CC). **(D–F)** KEGG Enrichment Bubble Plot of Differentially Expressed Genes. (The y-axis represents the pathway name, and the x-axis indicates the corresponding P-value for each pathway. The Rich factor is depicted by the color of the bubbles, with a larger value correlating with a more intense red color. The size of each bubble reflects the number of differentially expressed genes associated with that pathway.).

To uncover the biological processes and molecular pathways regulated by NOD1 in COPD, we implemented GO and KEGG enrichment analyses based on the identified DEGs. **Figures 5A–C** show bubble plot visualizations of the most significantly enriched GO terms (top 10 by P-value) across the biological process, cellular component, and molecular function categories for each group comparison. The shared biological processes across the three comparison groups include: system development, anatomical structure development, and multicellular organism development. The shared molecular functions include signaling receptor binding, while the shared cellular components include cell periphery, plasma membrane, and extracellular matrix. For KEGG pathway analysis, we selected the top 20 most enriched pathways and visualized them as bubble plots (**Figures 5D–F**, showing the KEGG enrichment results for each comparison group). DEGs across all comparison groups demonstrated shared enrichment in several pathways, including PI3K-Akt signaling, ECM-receptor interaction, and protein digestion and absorption. This suggests that NOD1 may influence the progression of COPD by regulating these signaling pathways.

### 3.8 Core gene and transcription factor analysis

**Figure 6:**
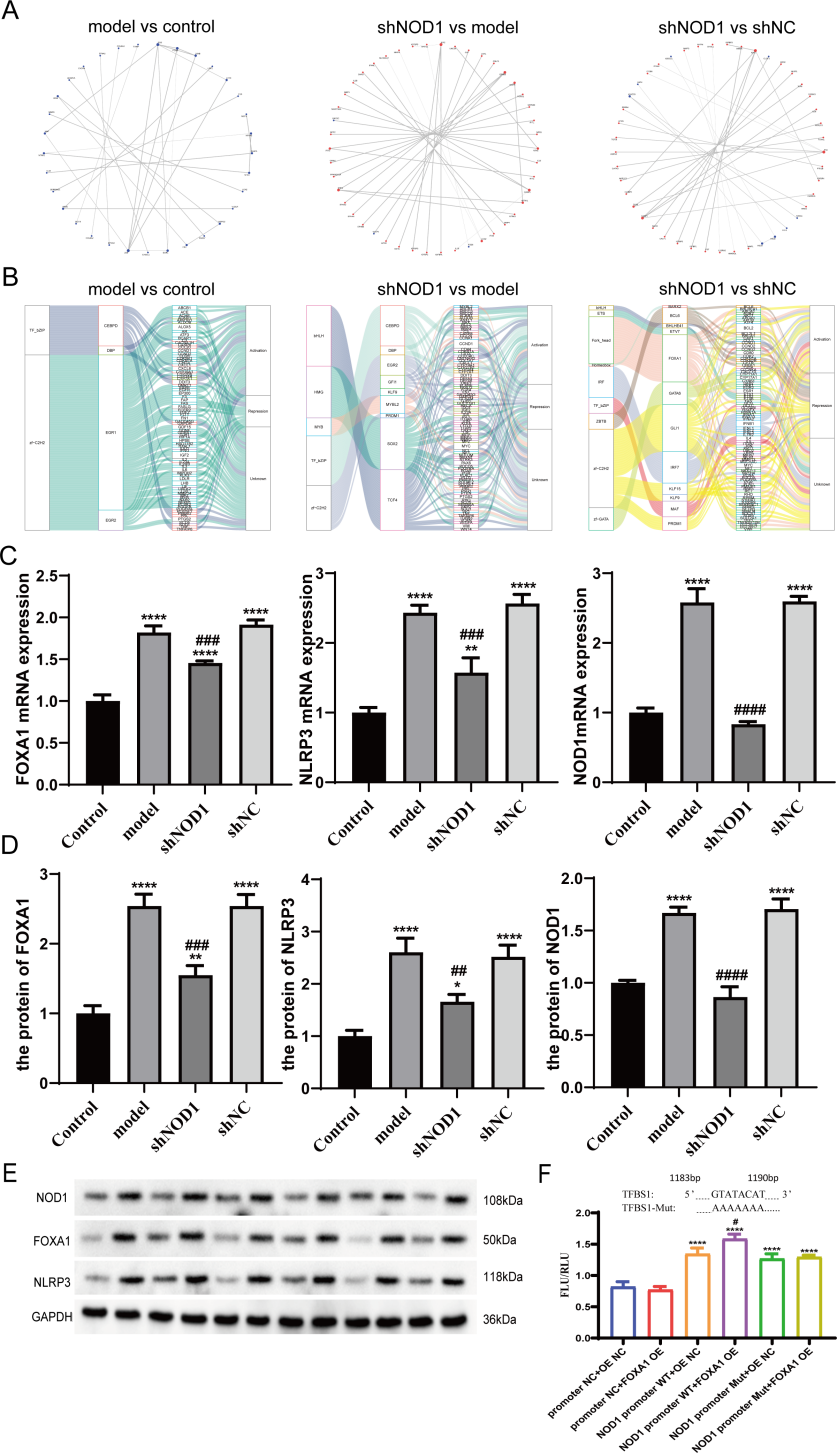
Core gene and transcription factor analysis and NOD1 activation of NLRP3 promotes pyroptosis in bronchial epithelial cells. **(A)** Differentially Expressed Gene Interaction Network(Each circle (node) in the network represents a gene; red circles indicate upregulated genes, and blue circles denote downregulated genes. Lines connecting these circles (edges) represent interaction relationships between genes. The network degree value of a gene is determined by the number of its interacting partners: a greater number of interacting genes corresponds to a higher network degree value. Accordingly, the size of each circle is proportional to its gene degree value, with larger circles representing higher degree values.) **(B)** Transcription Factor Target Sankey Diagram(From left to right, the columns represent transcription factor families, transcription factors, and target genes, respectively. The connecting lines (flows) between these columns illustrate the relationships among transcription factor families, transcription factors, and their target genes.) **(C–E)**; RT-PCR and WB analysis of NOD1, FOXA1 and NLRP3 levels in the Beas-2B cells **(F)**; Interaction between NOD1 and FOXA1**;**FOXA1 promoter luciferase activity was detected by dual-luciferase assays Data were expressed as mean ± SD, n = 3, ns, no significance, ****P < 0.001 NOD1 promoter WT+FOXA1 OE compared with NOD1 promoter WT+OE NC. **(C)** FOXA1mRNA expression **** p<0.0001 control vs model/shNC/shNOD1, ### p<0.001 model vs shNOD1, NLRP3 mRNA expression ** p<0.01 control vs shNOD1, **** p<0.0001 control vs model/shNC, ### p<0.001 model vs shNOD1, NOD1 mRNA expression **** p<0.0001 control vs model/shNC, #### p<0.0001 model vs shNOD1. **(D)** The protein of FOXA1 ** p<0.01 control vs shNOD1, ### p<0.001 model vs shNOD1, **** p<0.0001 control vs model/shNC the protein of NLRP3, * p<0.05 control vs shNOD1, ## p<0.01 model vs shNOD1, **** p<0.0001 control vs model/shNC the protein of NOD1, #### p<0.0001 shNOD1 vs model, **** p<0.0001 control vs model/shNC.

To pinpoint NOD1-related downstream genes involved in COPD, we analyzed protein-protein interactions (PPIs) among DEGs using STRING (http://string-db.org/). As shown in **Figure 6A**, we generated PPI networks for three comparison groups: model vs. control, shNod1 vs. model, and shNOD1 vs. shNC. The results revealed multiple death-related genes as downstream core genes of NOD1, including CASP1, NLRP3, IL-18, and IL-1β. Furthermore, we performed Transcription Factor Annotation using TRRUST2.0 for DEGs and visualized the transcription factor-target relationships through sankey diagrams (**Figure 6B**). The most enriched transcription factors in DEGs were identified as FOXA1, KLF9, and PRDM1, suggesting that NOD1 may regulate these transcription factors, thereby influencing pyroptosis. It is worth noting that although FOXA1 has been shown to be upregulated in COPD lung tissues (22), its particular impacts on COPD and the underlying mechanisms are yet to be clarified.

To confirm our sequencing data, we conducted RT-qPCR and Western blot validations of NOD1, FOXA1 and NLRP3 expressions. The model group had higher expressions of NOD1, FOXA1, and NLRP3 than the control group. In contrast, the shNOD1 group exhibited suppressed expression of these genes relative to the model and shNC groups (**Figures 6C–E**). These observations were in agreement with our sequencing results.

### 3.9 NOD1 mediated pyroptosis via FOXA1/NLRP3

In order to examine how NOD1 alters CSE+LPS-induced pulmonary pyroptosis in BEAS-2B cells, we utilized the JASPAR database (https://jaspar.elixir.io/) to predict transcription factor interactions. The results showed that FOXA1 can act as a transcription factor for NLRP3 (**Supplementary Table 8**). This suggests that NOD1 may regulate pulmonary pyroptosis through the FOXA1/NLRP3 pathway.

Dual luciferase assay results demonstrated that, compared to the NOD1 promoter WT + OE of negative control (NC), the expression levels significantly increased in the NOD1 promoter WT + FOXA1 overexpression (OE) group. This suggests an interaction between NOD1 and FOXA1 (**Figure 6F**).

**Figure 7:**
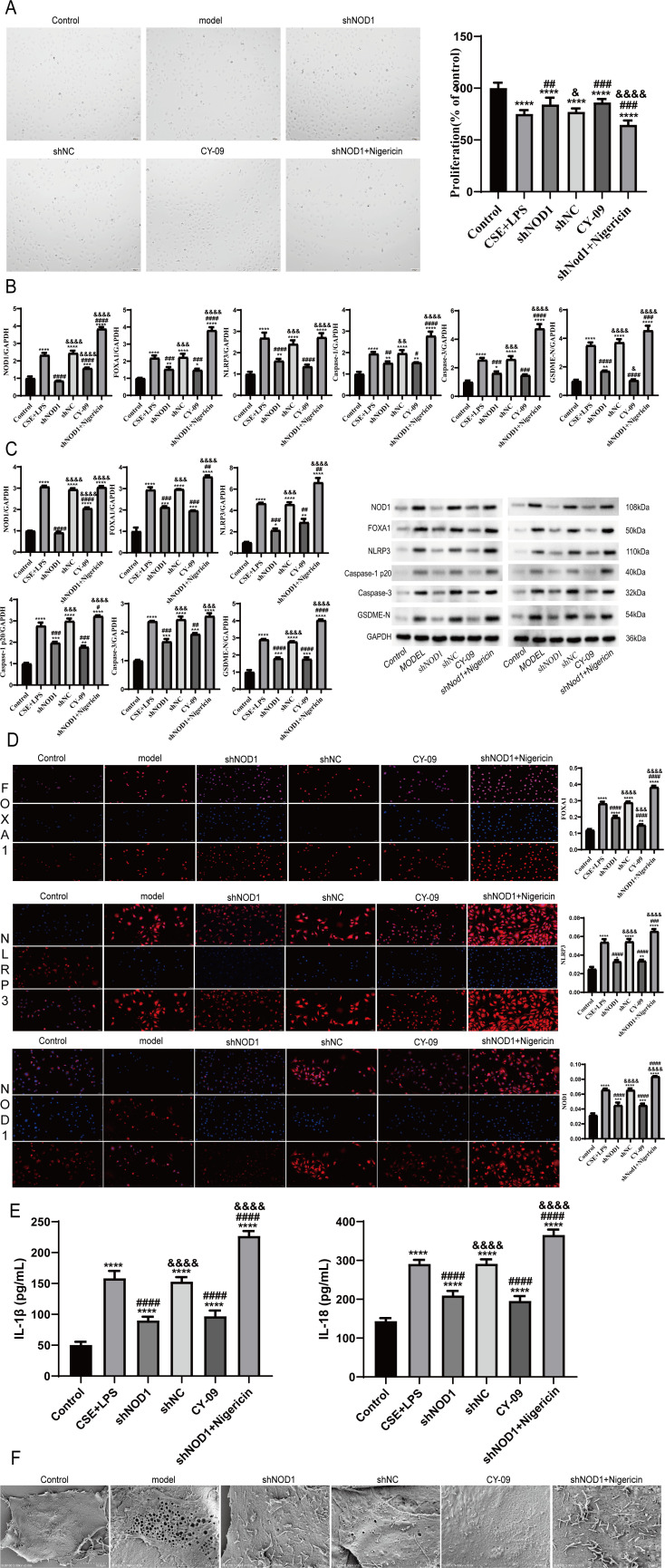
NOD1 activation of NLRP3 promotes pyroptosis in bronchial epithelial cells. **(A)** CCK assay for cell proliferation ability CCK8 ##p<0.01 model vs shNOD1, ###p<0.001 model vs CY-09/ shNOD1+Nigericin, **** p<0.0001 control vs model/shNC/shNOD1/CY-09/ shNOD1+Nigericin & p<0.05 shNOD1 vs shNC &&&& p<0.0001 shNOD1 vs shNOD1+Nigericin **(B, C)** RT-qPCR and Western blotting analyzed NOD1,FOXA1,NLRP3,Caspase-1,Caspase-3,GSDME-N expression in BEAS-2B cells after different treatments. For immunoblots, GAPDH was used as loading control. Data are representative of three independent experiments. Data are mean ± SD. ***p <0.001, *p <0.05; by one-way analysis of variance (ANOVA) by Student’s two-tailed t-test. **(D)** Fluorescence full scan of the expression of NOD1,FOXA1,NLRP3 cells after different treatments FOXA1 ****p<0.0001 control vs model/shNC/shNOD1/ shNOD1+Nigericin, **p<0.01 control vs CY-09, #### p<0.0001 model vs shNOD1/CY-09/ shNOD1+Nigericin, &&&&p<0.0001 shNOD1 vs shNC/ shNOD +Nigericin, &&&p<0.001 shNOD1 vs CY-09, NLRP3 ****p<0.0001 control vs model/shNC/ shNOD1+Nigericin, **p<0.01 control vs CY-09, *p<0.05 control vs shNOD1, ####p<0.0001 model vs shNOD1/CY-09, ###p<0.01 model vs shNOD1+Nigericin, &&&& p<0.0001 shNOD1 vs shNC/ shNOD1+Nigericin, NOD1 ****p<0.0001 control vs model/shNC/ shNOD1+Nigericin, ***p<0.001 control vs shNOD1/CY-09, ####p<0.0001 model vs shNOD1/CY-09/ shNOD1+Nigericin, &&&& p<0.0001 shNOD1 vs shNC/ shNOD1+Nigericin.. **(E)** ELISA results showing the expression levels of IL-1β and IL-18 in different groups IL-1β ****p<0.0001 control vs model/shNOD1/shNC/ CY-09/shNOD1+Nigericin, ####p<0.0001 model vs shNOD1/CY-09/ shNOD1+Nigericin, &&&&p<0.0001 shNOD1 vs shNC/ shNOD1+Nigericin, IL-18 ****p<0.0001 control vs model/shNOD1/shNC/ CY-09/shNOD1+Nigericin, ####p<0.0001 model vs shNOD1/CY-09/ shNOD1+Nigericin, &&&&p<0.0001 shNOD1 vs shNC/ shNOD1+Nigericin. **(F)** Representative scanning electron micrographs of BEAS-2B cells after treatment with different methods. Red arrows indicate formation of pyroptotic membrane pits and pores of varying size.(****p < 0.001 vs. Control group; ####p < 0.001 vs. Model group; &&&&p < 0.001 vs. sh-NC group.).

To further analyze the role of NOD1, we performed knockdown experiments in BEAS-2B cells. CCK-8 assays revealed reduced cell viability in the model group (**Figure 7A**). NOD1, FOXA1, NLRP3, Caspase-1, Cleaved Caspase-1, GSDMD, and GSDMD-N were found to be highly expressed in the model group, as confirmed by RT-qPCR and Western blot assays (**Figures 7B, C**). The increased levels of NOD1, FOXA1, and NLRP3 in the model group were validated by immunofluorescence (**Figure 7D**). Moreover, supernatant analysis unveiled increased concentrations of IL-18 and IL-1β levels in the model group (**Figure 7E**).

The model group cells showed typical pyroptotic morphology, such as chromatin condensation, mitochondrial swelling, and plasma membrane rupture, as in cellular pyroptosis (**Figure 7F**). All these results suggested a higher degree of pyroptosis in BEAS-2B cells in the model group. Next, BEAS-2B cell pyroptosis was substantially suppressed by NOD1 knockdown, which was demonstrated by the increased cell viability, reduced pyroptosis-associated gene expression, lower IL-18 and IL-1β levels, and the improved cellular morphology (**Figures 7A–F**). In contrast, NLRP3 overexpression was effective to overcome the inhibitory effect of NOD1 knockdown on cellular pyroptosis (**Figures 7A–F**).

These findings, as a whole, indicate that NOD1 knockdown suppresses cell pyroptosis in CSE+LPS-stimulated cells through FOXA1/NLRP3.

## 4 Discussion

COPD ranks among the most serious chronic diseases worldwide, involving irreversible airflow limitation and symptoms primarily due to airway and alveolar damage from chronic inhalation of harmful agents like CS (23). COPD has different phenotypes, such as chronic bronchitis and emphysema, so it presents with diverse clinical manifestations and complications, including chronic respiratory failure and cardiovascular diseases (24, 25). Worsening COPD is commonly associated with diminished quality of life and a marked increase in both morbidity and mortality, which has prompted the need to improve our understanding and management strategies to mitigate its impacts (26).

We examined the role of NOD1 in controlling the expressions of FOXA1 and NLRP3 in COPD in this study. Sophisticated technologies were used to support the execution of this study, such as RNA-Seq transcriptomic analysis and mechanistic validation tools, which assisted in tracing the molecular mechanisms of NOD1 regulation of cell pyroptosis *via* FOXA1 and NLRP3, as well as the inflammatory processes that contribute to COPD development (12) (27). RNA-Seq analysis revealed that differentially expressed genes were significantly enriched in the PI3K-Akt, ECM-receptor interaction, and inflammation-related pathways, all of which are closely associated with the NOD1-FOXA1-NLRP3 axis. As a key downstream regulatory pathway of NOD1, PI3K-Akt signaling can modulate FOXA1 transcriptional activity and influence NLRP3 expression, thereby facilitating inflammasome assembly and pyroptosis (28, 29). Additionally, the upregulation of extracellular matrix–related genes suggests ongoing airway remodeling and inflammatory injury, processes that may also be driven by NOD1-mediated chronic inflammatory signaling (30). Collectively, these findings indicate that NOD1 amplifies the inflammatory and cell death responses in COPD by integrating multiple signaling pathways, providing a molecular explanation for disease progression (14, 15). In house dust mite-sensitized asthmatic mice, NOD1 enhances the response of T helper 2 (Th2) (31). In the meantime, NOD1 engages in antifungal host defense by affecting Dectin-1 expression (32). NOD1, an intracellular pattern recognition receptor, is highly expressed in inflammatory cells in the lung. It identifies bacterial peptidoglycan fragments and activates downstream TLR4/NF-κB signaling pathways, thereby amplifying inflammatory signaling pathways through increased IL-6 and TNF-α release and worsening the impairment of lung functions and tissue destruction caused by CS (19). Analysis of bronchial mucosa from COPD patients has revealed heightened NOD1 expression, particularly in those with rapidly worsening lung function, and this expression is positively linked to the rate of lung deterioration and smoking history, indicating that NOD1 may play a role in COPD progression (33). These results indicate that NOD1 is a promising new therapeutic target in the management of COPD, and that the complexity of the molecular pathways of this debilitating condition deserves further elucidation (34) (35).

NOD1 senses bacterial-associated molecular patterns and activates downstream inflammatory signaling pathways. FOXA1 is an innovative transcription factor critical for pulmonary epithelial differentiation and the modulation of inflammation (36). NLRP3 inflammasome engagement activates caspase-1, which processes IL-1β and IL-18 and cleaves GSDMD to form membrane pores ultimately causing pyroptosis (37, 38).In addition to NLRP3, other inflammasome sensors such as AIM2 and NLRC4 have also been implicated in chronic pulmonary inflammation and infection (39, 40). However, transcriptomic analyses and validation experiments in this study revealed no significant expression changes in these molecules, suggesting that they may not serve as principal pro-inflammatory drivers in the present model. Nonetheless, their potential regulatory roles in COPD progression under various pathological conditions or stimuli warrant further investigation.

It has been proposed that the NLRP3 inflammasome can be assembled and activated under the regulatory influence of NOD1 through the regulation of FOXA1 expression or activity. FOXA1 is capable of regulating the expression of NLRP3 and other inflammatory genes, which induces an amplification effect of a signaling cascade. NOD1 indirectly regulates the transcriptional activity of NLRP3 through FOXA1, while overexpression of NLRP3 can directly restore inflammasome activation. This observation suggests that NLRP3 serves as a critical downstream node within the NOD1-FOXA1 signaling axis. Upon knockdown of NOD1, enhanced FOXA1 transcriptional activity suppresses NLRP3 expression and inflammasome activation; conversely, exogenous overexpression of NLRP3 bypasses this transcriptional inhibition, directly promoting Caspase-1 cleavage and GSDMD-N formation, thereby reactivating pyroptosis. Furthermore, previous studies have demonstrated that NOD1 can regulate the expression of inflammation-related genes via the NF-κB or PI3K-Akt pathways, indicating that indirect signaling intermediates may exist within this feedback circuit (41, 42). Taken together, these findings reveal a multilayered regulatory relationship among NOD1, FOXA1, and NLRP3, collectively determining the strength and persistence of pyroptotic responses in COPD (11, 27).

It should be noted that in this study, NOD1 activation was not induced by exogenous ligands, but rather driven by endogenous stress signals elicited by CSE and LPS exposure. This non-specific mode of activation more closely reflects the actual pathological environment of COPD, where chronic cigarette smoke exposure and bacterial components synergistically lead to sustained activation of NOD1-mediated inflammatory signaling.

NOD1 has been shown to engage the NF-κB and MAPK signaling pathways upon activation, thus supporting the transcriptional regulation of FOXA1 and NLRP3 (43) (38). This axis is dynamically regulated to define the degree of pyroptosis in lung epithelial cells and macrophages and influences COPD inflammatory and tissue injury. Current findings suggest that NOD1 may modulate the transcriptional activity of FOXA1 by activating the PI3K-Akt and NF-κB signaling pathways, thereby weakening FOXA1-mediated repression of the NLRP3 promoter. Downregulation of FOXA1 releases its transcriptional inhibition on core components of the inflammasome, resulting in increased NLRP3 expression, enhanced Caspase-1 activation, and GSDMD cleavage, ultimately triggering pyroptosis and the release of inflammatory cytokines. This hierarchical regulatory mechanism provides a molecular explanation for how NOD1 signaling amplifies the inflammatory response in COPD (19). As a prototypical pioneer transcription factor, FOXA1 plays a critical role in maintaining airway epithelial cell differentiation and homeostasis, and it has been shown to suppress the transcription of various inflammation-related genes by modulating chromatin accessibility (36). Previous studies have demonstrated that downregulation of FOXA1 impairs the anti-inflammatory capacity of the epithelial barrier (43), thereby amplifying inflammatory signaling cascades. In light of our current findings, it can be inferred that NOD1 activation may promote inflammatory cell pyroptosis by inhibiting the expression or function of FOXA1, thereby relieving its transcriptional repression of NLRP3 and other inflammatory genes.

The results of the present research contribute to the overall comprehension of the molecular pathways underlying COPD, namely, by regulating NOD1, FOXA1, and NLRP3. However, previous studies have demonstrated that ferroptosis (44)and necroptosis (45) are also involved in the pathogenesis of COPD. We speculate that there may be crosstalk among these pathways in cellular stress responses, oxidative injury, and immune regulation (46).The identified augmented expression of NOD1 in COPD cells and its connection to the regulation of FOXA1 and NLRP3 expression disclose a pathway that has not been thoroughly investigated and could be functionally linked to the progression of inflammatory processes that characterize this ailment. The clarification of this association enlightens us of the complexity of the interactions of these molecular actors, which can be a basis of future therapeutic approaches towards lessening the inflammatory response seen in COPD. This pathway in COPD may have implications in other inflammatory diseases, in which NOD1 signaling is highly important and, therefore, exerts a wider impact on the area of inflammatory disease management (11).

Alongside the molecular mechanisms, the findings of this work also emphasize the prominent roles of gene activity and cell behavior in COPD. The expressions of the main genes related to pyroptotic processes, such as CASP1, NLRP3, IL-18, andIL-1β, highlight the role of NOD1 and FOXA1 in controlling the production of inflammatory reactions and pyroptosis. The changes in cell viability in response to NOD1 knockdown also help understand what adaptive mechanisms are implemented in response to cell stress *via* NOD1 knockdown, which provides clues to how such mechanisms can be controlled to increase cell survival in chronic inflammation. The insights into the cellular responses to NOD1 signaling and the downstream effects on pyroptosis would help appreciate the complexities of the lung tissue homeostasis and the prospects of developing specific interventions that can potentially enhance the outcomes of patients with COPD and other disease types (47).

The immune pathways that are explained in this paper also add to the accumulating literature that emphasizes the potential regulatory influence of NOD1 on inflammatory activity. Observed elevations in IL-18 and IL-1β following NOD1 stimulation suggest a direct contribution to the disrupted immune regulation seen in COPD patients. This is an indication that the targeting of NOD1 can not only reduce the localized inflammation of the lungs but also cause systemic effects that regulate more immune responses. Moreover, the prospect of NOD1-related immune pathways being used as biomarkers of disease severity or therapeutic efficacy is a viable direction of future research with the aim of refining the accuracy of COPD treatment (33).

Although the current study offers significant information on the role that NOD1 plays in the development of COPD, it is not without limitations. A limitation is that there is no validation *in vivo*, which decreases the translational potential of our results. The article mainly uses a small sample size in the experimental design, which can influence the strength and applicability of the measured observations. Moreover, the absence of a clinical correlation analysis limits us to making final conclusions about clinical relevance of the identified molecular mechanisms in COPD patients. Such limitations highlight the importance of future studies with the involvement of larger cohorts and different experimental models to validate our results and explain the complex interaction between NOD1, FOXA1, and NLRP3 in COPD.

To sum up, this paper clarifies the importance of NOD1 in the regulation of FOXA1 and NLRP3, which eventually affect cell pyroptosis and the pathogenesis of COPD. Our results indicate the interdependent relationships between molecular pathways and signaling pathways that support COPD progression, which can be subjected to therapeutic intervention. Through the regulatory interactions of NOD1 with its downstream effectors, we pave the way for future studies with the objective of developing specific therapies to alleviate inflammatory mechanisms involved in COPD. This work not only deepens our understanding of the disease but also create opportunities to develop new approaches to treatment that might have a great influence on patient outcomes.

## Data Availability

The data presented in the study are deposited in the Sequence Read Archive (SRA) repository, accession PRJNA1445891.
