# Supplementary material for: NOD1 modulates chronic obstructive pulmonary disease progression via FOXA1/NLRP3-mediated regulation of pyroptosis
Source: Front Immunol. 2026 Apr 17;17:1800191. doi: 10.3389/fimmu.2026.1800191 (PMC13134683; doi:10.3389/fimmu.2026.1800191)
Supplement: Supplementary file 1 [file DataSheet1.pdf]

Supplementary Table 1 AAV-LungM3 specifically targeting the lungs

Plasmid	serotype	lentivirustitre （Tu/ml）
AVV-Control	LungM3-targeted delivery to lung tissue	1.21E+13
AVV-Ininterference1		1.81E+13
AVV-interference2		1.67E+13
AVV-interference3		9.31E+12

Supplementary Table 2 Sequences of shRNAs

Plasmid	VectorStructure	shRNAsequence(5-3’)
Control	pCLenti-U6-shRNA(NC)-CMV-Puro-WPRE	TTCTCCGAACGTGTCACGT
interference plasmid1	pCLenti-U6-shRNA1（NOD1）CMV-Puro-WPRE	GAAAGTCAACCAGACGTTAAA
interference plasmid2	pCLenti-U6-shRNA2（NOD1）CMV-Puro-WPRE	CGAAGAGCTGACCAAATACAA
interference plasmid3	pCLenti-U6-shRNA3（NOD1）CMV-Puro-WPRE	GTGGACAACTTGCTGAAGAAT

Supplementary Table 3 shNOD1-expressing AAV vectors for NOD1 knockdown

Plasmid	VectorStructure	shRNAsequence(5-3’)
Control	pAAV-U6-shRNA(NC)-CMV-EGFP-WPRE	TTCTCCGAACGTGTCACGT
interference plasmid11	pAAV-U6-shRNA1(Nod1)-CMV-EGFP-WPRE	CAGGGCCAGTCTTACGAATTT
interference plasmid2	pAAV-U6-shRNA2(Nod1)-CMV-EGFP-WPRE	CGTGACGTTCCTGGGTTTATA
interference plasmid3	pAAV-U6-shRNA3(Nod1)-CMV-EGFP-WPRE	TGAGGAACTGACCAAGTATAA

Supplementary Table 4.   Lentivirus Vector Map

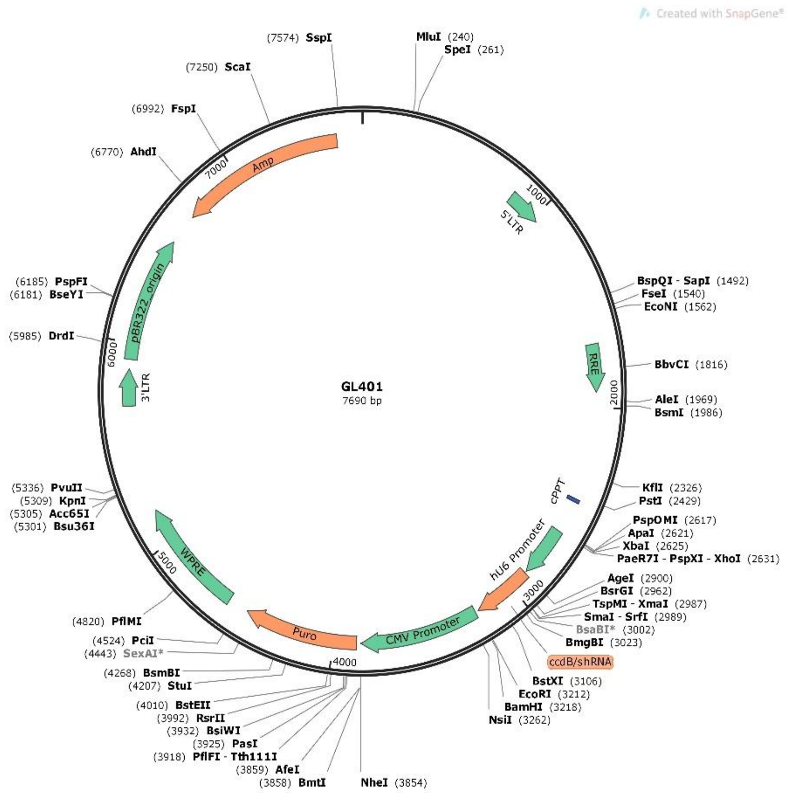

Supplementary Table 5   Lentivirus Vector

Plasmid	lentivirustitre（Tu/ml）
LV-Control	4.83E+08
LV-Ininterference1	3.61E+08
LV-interference2	5.00E+08
LV-interference3	3.30E+08

Supplementary Table 6 The primer sequence used in the qRT-PCR

Human	Forwardprimer(5’-3’)	Reverseprimer(5’−3’)	Fragmentlength
Nod1	CCACAGTGAGATGGAAAT	TGGAGCAAGTAGAGGAAG	258bp
FOXA1	CGCCACTCGCTGTCCTTC	GGGTCCTTGCGGCTCTCA	236bp
NLRP3	AGAGGAGTGGATGGGTTT	CGTGTGTAGCGTTTGTTG	168bp
Caspase-3	AAACCTCAGGGAAACATT	CTCAGAAGCACACAAACA	144bp
Caspase-1	GAAGGACAAACCGAAGG	GGAAGAGCAGAAAGCGA	181bp
GSDME-N	AGTTTTTATCCCTCACCC	ATTTATTGTTCTCTCGGC	260bp
GAPDH	GGAGCGAGATCCCTCCAAAA	GGCTGTTGTCATACTTCTCATGG	197bp

Supplementary Table 7 Filtering of raw transcriptome data

Sample	Raw_reads	Clean_reads	Clean_bases	Error(%)	Q20(%)	Q30(%)	GC(%)
control-1	48439904	48137462	7.19G	0.02	98.03	94.26	50.69
control-2	46067102	45771814	6.84G	0.02	98.09	94.44	51.01
control-3	54640664	54275874	8.11G	0.02	98.08	94.4	50.83
model-1	50170248	49878002	7.46G	0.02	98.09	94.4	50.94
model-2	53680120	53337510	7.97G	0.02	98.19	94.67	50.95
model-3	50269156	49959972	7.48G	0.03	97.99	94.13	50.17
shNC-1	57175260	56789596	8.5G	0.03	97.93	94.02	50.99
shNC-2	52968932	52614682	7.87G	0.03	97.89	93.9	50.49
shNC-3	51171828	50811980	7.6G	0.02	98.06	94.37	50.42
shNod1-1	51835306	51512564	7.71G	0.02	98.1	94.42	50.42
shNod1-2	47382664	47070084	7.05G	0.03	97.93	94.03	50.39
shNod1-3	50518974	50224114	7.52G	0.02	98.07	94.31	50.99

Supplementary Table 8 Dual luciferase assay predicted binding site

NOD1[Human]：

Matrix ID	Name	Score	Relative score	Sequence ID	Start	End	Strand	Predicted sequence
MA0148.5	MA0148.5.FOXA1	9.793092	0.89563733	NC_000007.14:c30480784-30478684	1181	1188	-	GTATACAT
MA0148.5	MA0148.5.FOXA1	9.793092	0.89563733	NC_000007.14:c30480784-30478684	1183	1190	+	GTATACAT
MA0148.5	MA0148.5.FOXA1	7.898545	0.85413164	NC_000007.14:c30480784-30478684	888	895	-	ATAAACAG
MA0148.5	MA0148.5.FOXA1	6.783358	0.82970005	NC_000007.14:c30480784-30478684	530	537	-	GAAAACAG
MA0148.5	MA0148.5.FOXA1	6.7041397	0.82796454	NC_000007.14:c30480784-30478684	512	519	+	TTAAACAC
MA0148.5	MA0148.5.FOXA1	5.802777	0.8082176	NC_000007.14:c30480784-30478684	1605	1612	-	GTAATCAA
MA0148.5	MA0148.5.FOXA1	5.5725174	0.80317295	NC_000007.14:c30480784-30478684	24	31	-	TTACACAT

NLRP3[Human]：

Display 10 profiles Filter:

Matrix ID	Name	Score	Relative score	Sequence ID	Start	End	Strand	Predicted sequence
MA0148.5	MA0148.5.FOXA1	9.377762	0.8865382	NC_000001.11:247414077-247416177	314	321	-	GTAAAAAT
MA0148.5	MA0148.5.FOXA1	9.021854	0.8787411	NC_000001.11:247414077-247416177	36	43	-	CTAAACAT
MA0148.5	MA0148.5.FOXA1	6.581715	0.8252825	NC_000001.11:247414077-247416177	510	517	+	CTAAACAA
MA0148.5	MA0148.5.FOXA1	6.5407476	0.824385	NC_000001.11:247414077-247416177	1143	1150	-	GAAAACAC
MA0148.5	MA0148.5.FOXA1	6.329998	0.81976795	NC_000001.11:247414077-247416177	1740	1747	-	GTAAAGAA
MA0148.5	MA0148.5.FOXA1	5.8508625	0.809271	NC_000001.11:247414077-247416177	177	184	+	GCCAACAT
MA0148.5	MA0148.5.FOXA1	5.8508625	0.809271	NC_000001.11:247414077-247416177	756	763	-	GCCAACAT
